# Retrospective Experiences of First-Episode Psychosis Treatment Under Open Dialogue-Based Services: A Qualitative Study

**DOI:** 10.1007/s10597-021-00895-6

**Published:** 2021-09-22

**Authors:** Tomi Bergström, Jaakko Seikkula, Juha Holma, Päivi Köngäs-Saviaro, Jyri J. Taskila, Birgitta Alakare

**Affiliations:** 1 Department of Psychology, University of Jyväskylä, Jyvaskyla, Finland; 2 Department of Psychiatry, Länsi-Pohja Hospital District, Kemi, Finland

**Keywords:** Family therapy, First person accounts, Long-term follow-up, Need-adapted approach, Schizophrenia

## Abstract

Open Dialogue (OD) is an integrated approach to mental health care, which has demonstrated promising outcomes in the treatment of first-episode psychosis (FEP) in Finnish Western Lapland region. However, little is known how treatment under OD is retrospectively experienced by the service users themselves. To address this, twenty participants from the original Western Lapland research cohort diagnosed with psychosis (F20–F29) were asked about their treatment of FEP, initiated under OD 10–23 years previously. Thematic analysis was used to explore how the treatment was experienced. Most participants viewed network treatment meetings as an important part of their treatment, as they enabled interactions with other people and the chance to go through difficult experiences. A minority of the participants had mixed experiences regarding family involvement and immediate home visits. OD may have the potential to promote therapeutic relationships, but replications from other catchment areas are needed.

## Introduction

As mental health research and practice moves beyond a focus on group-level symptom-reduction models, an increasing emphasis has been placed on service users’ personal experiences of recovery and on other existential values (e.g. Frost et al., 2017; van Os et al., 2019). Open Dialogue (OD) is one example of an approach whose primary goal encompasses service-user involvement in shared decisions and meaning-making processes. In OD, joint network treatment meetings take place in response to acute psychological crises (Seikkula et al., 2011). These meetings are often organized at the patient’s home or in another safe environment of the patient’s choice. The aim is to immediately gather all relevant people together to create a shared understanding of each situation within reciprocal dialogues. Thus, within the meetings every perspective is accepted unconditionally and all interpretations and treatment decisions are made together with the patients and their close networks. The main task for professionals is not to determine the specific diagnosis and treatment, but rather to create a safe space where everyone can be heard (Seikkula et al., 2006). The promotion of dialogue is understood to increase the sense of agency in the lives of patients and their families, while simultaneously enabling a more flexible and individualized integration of existing services and methods in efforts to address a difficult life situation in a need-adapted manner (Bergström, 2020).

OD is based on naturalistic research work conducted in Finland since the 1970s, within which the primary aim has been to develop need-adapted practices within public mental health care services (Alanen, 2009). In one region in particular, consisting of the western parts of Finnish Lapland, the entire mental health care system was gradually reorganized in such a way that a dialogical and network-oriented response to crisis was possible for all psychiatric patients in the region (Seikkula et al., 2011). In parallel with the regional development of services in Western Lapland, several naturalistic research projects evaluated the effectiveness of the approach in the treatment of first-episode psychosis (FEP) (Seikkula et al., 2003, 2006, 2011). The projects included all FEP patients in the Western Lapland region at the time when the practice was implemented (Seikkula et al., 2011). Historical comparisons showed promising results relating to the patients’ functional outcomes (Seikkula et al., 2003, 2006, 2011). More recent register-based studies have confirmed the long-term stability of the good outcomes (Bergström et al., 2018), and also their superiority to standard treatment of FEP (Bergström, 2020; Bergström et al., 2018). Subsequently, promising results have been reported in various implementation projects conducted outside the Western Lapland region (Bouchery et al., 2018; Buus et al., 2017, 2019; Gordon et al., 2016; Grano et al., 2016), although more robust evidence on the generalizability and transferability of the approach is still needed (Freeman et al., 2019).

There have also been reports on how patients and their family members experienced OD at early phases of its implementation. According to these, service users felt that under OD they had been better listened to and understood (Florence et al., 2021; Gidugu et al., 2021; Hendy et al., 2020; Tribe et al., 2019; Twamley et al., 2020), notably in comparison with their earlier care experiences, even if a minority had also found the network treatment meetings to be emotionally overwhelming and strange (Tribe et al., 2019). In our earlier study (Bergström et al., 2019) it was found that among those service users who participated in the original OD projects in Western Lapland, the mental health treatment itself was not viewed as the most central element in their gradual recovery from psychosis. In fact, other life areas were regarded as more important, reflecting a general tendency to associate the psychotic crisis with cumulative life adversities (Bergström, 2020).

The studies so far have not conducted qualitative assessments of how the mental health treatment of FEP under OD-based services was retrospectively experienced over the longer term, both in general and in the original Western Lapland research cohort in particular. This study aimed to address this lack of knowledge by asking 20 participants from the Western Lapland research cohort about their experiences of FEP treatment, initiated under OD-based services 10–23 years previously.

## Methods

### Study Design

The participants for this qualitative research were recruited from Western Lapland research cohorts, which included all persons (*N* = 108) whose first treatment contact with non-affective psychosis (ICD-10-codes F20-F29) occurred in the Western Lapland region during the time of the three OD research phases (1992–1993, 1994–1997, and 2003–2005). One goal of the project was to study how mental health crisis diagnosable as a psychosis was narrated as a part of a life story (Bergström et al., 2019). The other aim was to explore participants’ experiences on the mental health treatment applied in their crisis. This latter aim was the main focus of the study reported here.

The chance to participate was offered via letters to all cohort members who were living within a reasonable distance (500 km) from the catchment area (*N* = 77). The letter invited the candidates to talk about their life-course, and to give feedback on the network-oriented mental health treatment they had received. In addition to the letter, the local healthcare staff in Western Lapland healthcare districts were asked to directly invite those cohort members who were still receiving some form of mental health care in the Western Lapland region (*N* = 18; 23% of all invited cohort members) in the year 2016. Out of the 77 persons invited, 14 (18%) declined, while 21 (27%) expressed their willingness to participate. The remainder (55%) did not react, and one retracted participation.

More detailed information on the cohort and on the data collection is presented elsewhere (Bergström, 2020).

### Interviews

All the interviews were conducted by the first author, usually accompanied by a co-interviewer from the hospital staff or the research group. The interviews took place in the participant’s home or in the nearest outpatient clinic, according to the participant’s own decision.

A semi-structured life-story interview frame was used during the interviews (Bergström et al., 2019). The frame was supplemented with open-ended questions on mental health crises and experiences of mental health treatment.

At the beginning of the interview all the participants were encouraged to relate their life stories as precisely as they could. The participants were then asked to say more about the experiences that led to initial treatment contact in the Western Lapland region. At the end of each interview, all the participants were encouraged to give more detailed information on their mental health treatment, indicating the aspects they had found helpful and/or unhelpful in their initial mental health treatment in Western Lapland, and what could have been done differently.

All the participants were aware that first author was not part of their treatment team, and all the participants had the opportunity to give feedback on their treatment, without the presence of current and/or former treatment team members.

The interviews were transcribed verbatim by the first author. The average length of the interviews was 97 min (min = 52, max = 157).

### Analyses

An inductive semantically-based thematic analysis (Braun & Clarke, 2006) was used to explore how treatment was experienced. At the first phase of the analysis initial codes are generated by detecting central features of the data across the entire data set. At next strep codes are collated into potential themes, gathering all data/content relevant to potential themes. Then themes are checked in relation to the coded extracts and the entire data set. At the final steps specifics of each theme are refined and themes are named.

The initial coding of the transcriptions was conducted as a part of an earlier study (Bergström et al., 2019). To address the main goal of this study, the codes relating to treatment of FEP were identified from the transcriptions. The themes were created by reviewing codes, and by identifying similarities and overlaps between codes. Throughout the process, the validity of the themes was reviewed against the raw data. The credibility of the themes was discussed with the entire research group. The analysis continued until no new themes emerged.

### Ethical Considerations

The research protocol was reviewed and approved by the North Ostrobothnia Hospital District Ethical Committee. All the participants completed written informed consent forms, in which they gave permission to use the information obtained via interviews. All the participants were given the opportunity to continue discussions afterwards with experienced clinicians.

## Results

### Participants

The clinical and demographical information on participants is presented in Table 1. As compared to the remainder of the cohort, there were indications that the participants had, at a general level, suffered more severe symptomatology, probably due the more direct recruitment of those who were still under treatment in Western Lapland region.

**Table 1:** Demographic and clinical characteristics of the interviewees, and of the rest of the cohort

	Interviewees (N = 20)		Refused/no reaction (N = 57)		Total cohort (N = 108)	
	N	%	N	%	N	%
*Baseline characteristics*^a^						
Age (M ± SD)	25 ± 9		25 ± 7		25 ± 7	
Male	12	60	24	42	62	57
Unemployed/passive	5	25	10	17	24	22
Civil status, single	13	65	43	75	82	76
Living alone	5	25	20	35	38	35
Treatment group (inclusion years)^b^						
API (1992–1993)	10	50	15	26	37	34
ODAP (1994–1997)	5	25	27	47	47	43
ODAP-II (2003–2005)	5	25	15	26	24	23
Diagnosis						
Acute/transient	9	45	24	42	49	45
Schizophreniform	4	20	11	19	22	20
Schizophrenia	7	35	22	39	38	35
Antipsychotics in first year	7	35	11	19	22	20
Hospital admission in first year	12	60	24	42	43	40
*Treatment during follow-up*^c^						
Hospital days (M ± SD)	100 ± 200		62 ± 123		63 ± 131	
Hospital admissions (M ± SD)	4 ± 5		3 ± 4		3 ± 5	
Antipsychotics	13	65	31	54	59	55
*Characteristics at the time of the interview*^c,d^						
Age (M ± SD)	45 ± 11		44 ± 9		45 ± 9	
Disability allowance	8	40	18	32	32	33
Antipsychotics	7	35	18	32	32	33
Treatment contact	7	35	16	28	26	27

*M* mean, *SD* standard deviation, *API* Acute Psychosis Integrated study, *ODAP* Open Dialogue in Acute Psychosis study

^a^More detailed information on baseline characteristics, treatment groups, and diagnostic procedures are described elsewhere (Bergström, 2020)

^b^Inclusion period of three Open Dialogue research projects

^c^Data were gathered as part of long-term register-based follow-up (Bergström et al., 2018)

^d^Only people still alive at the end of the follow-up

### Overview of Findings

On the basis of the analyses we identified nine subthemes (see Table 2). These were further organized into three main domains according to their thematic content: (1) the importance of the relationships in the context of mental health care, (2) ambivalence related to the immediate response and teamwork*,* (3) ambivalence related to the hospitalization and medication. Note that none of the themes was presented in an exclusive manner.

**Table 2:** Three thematic domains and sub-themes

Domain	Sub-theme
1. Importance of the relationships in the context of mental health care	1.1. Importance of the therapeutic relationship, *N* = 4 1.2. Treatment meetings as a positive experience, *N* = 12 1.3. Opportunities for social participation, *N* = 4
2. Ambivalence related to the immediate response and teamwork	2.1. Confusion relating to immediate home visits, *N* = 3 2.2. The role of the family in network treatment meetings, *N* = 3 2.3. Too many people involved in the treatment, *N* = 3 2.4. Wish for direct advice *N* = 2
3. Ambivalence related to hospitalization and medication	3.1. Hospital as a scary environment *N* = 2 3.2. Ambivalence related to medication, *N* = 4

### Importance of the Relationships in the Context of Mental Health Care

In line with OD, all the participants had had at least one family network treatment meeting immediately on treatment contact. Overall, 12 (60%) of the participants indicated that the network treatment meetings themselves had formed an important element in their mental health treatment.

When that treatment started and people came to visit once a week or even more, I soon noticed that I started to look forward to the next session and the opportunity to talk about these issues. Little by little things started to unwind and eventually I was able to move on in my life. [Paul, over 40 years old at the time of the interview]

Four (20%) participants specified the importance of simply having someone who came and showed an interest. Others emphasized the possibility of freely discussing their difficult experiences.

It was important for me just to notice that there were at least some people who were interested in me, and I felt that they were genuinely worried about my situation. [Robert, 20–40 years old]

The best part of the treatment was just the opportunity to go through those difficulties in those meetings. [Audrey, over 40 years old]

It helped when people were just present and when they didn’t judge you or anything like that. I think it was the most important part of that treatment. [Peter, 20–40 years old]

Some participants indicated that the important part of their treatment was not any given method per se, emphasizing rather the opportunity for socialization with other people.

Those groups gave me the chance just to be with other people. It felt kind of a good thing back then. [Thomas, over 40 years old]

Actually I’d kind of like to be in hospital. If they had asked me, it (hospital treatment) could have lasted even longer. At least there I wasn’t alone. [Laura, over 40 years old]

### Ambivalence Related to the Immediate Response and Team Work

A minority of the participants had ambivalence experiences regarding factors in their mental health care that can be interpreted as characteristic of OD. Three (15%) of the participants indicated that at the start of their treatment they had been surprised when people suddenly came to their house, but that eventually they had come to view that aspect in a positive light.

They (the hospital staff) suddenly came to our house and tried to discuss things with me and my parents. They also asked whether I would need some medication and I just said I don’t need anything. Well, it’s ok that they came, but for me it was just time that helped. [Amy, over 40 years old]

I was quite surprised when people suddenly came to our house and at the time I didn’t know whether it was a good or bad thing. Now I think it was a good thing. [Laura, over 40 years old]

Three participants (15%) felt that sometimes there had been too many staff and/or network-members participating in the meetings.

Sure it felt good that someone was interested in me. But often there were too many people in those meetings, and I remember it was kind of drag to go there, but maybe those (meetings) still somehow helped me to get over the worst of it. [John, over 40 years old]

I found it difficult to talk in the meetings. Maybe there were too many people. And as my husband was there as well, I felt that I couldn’t really express myself, as communication with him was my main problem in the first place. [Sarah, over 40 years old]

Even though all the participants had at least one family member who had participated in their treatment meetings, only three (15%) of the participants gave more specific feedback on the role of family members in the meetings. One thought that for him it had been the most important factor in his treatment, while two others mentioned this aspect as a negative factor overall.

Those meetings gave us opportunity to discuss those difficulties and it really was a turning point. You know, we (family members) really didn’t talk about those issues outside of those meetings. Back then our situation was so challenging it was a good thing that we had this opportunity to go through it. [Jack, over 40 years old]

I felt that it hadn’t worked out in our situation. I mean the thing that a group of people just come to our house and tried to discuss my feelings together with my husband. I just felt that it made him even more angry. I was also so ashamed; wondering what the neighbours would think! [Sarah, over 40 years old]

Two (10%) participants expressed the wish that someone would have given them more direct advice during their treatment.

I just wished that someone among the workers would tell me straight away what to do, and just put some sense in my head. But then again, I’m fairly certain that at the end of the day that wouldn’t have made any difference. [Andy, over 40 years old]

It would have been more helpful if someone had given me some kind of specific method that can be used in those difficult situations. [John, over 40 years old]

### Ambivalence Related to the Hospitalization and Medication

Twelve (60%) participants were hospitalized to a regional psychiatric hospital during their first treatment year. Two of them said that the psychiatric ward was rather a frightening environment, especially at the start of the treatment, at a time when they were already confused due to the psychosis.

The hospital was quite a scary place. I was so messed up and I remember that I was really afraid of those other patients as well. [Andy, over 40 years old]

The first time in the hospital was really quite a scary experience. I was so confused, I really didn’t know what was going on or where I was. It was kind of traumatic. But then again I don’t know if there’s anything else that could have been done. I was so out of control. [Paul, over 40 years old]

Out of the seven participants who received antipsychotics in their first treatment year, three viewed the antipsychotic medication as useful, while two expressed the notion that it had caused more harm than good.

Sure those meds (antipsychotics) helped me to calm down and I didn’t fool around so much anymore. I mean I wasn’t so out of control anymore. But the side effects were awful. I felt that they somehow flattened –, no, I mean that they completely knocked out all my thoughts, my dreams and goals. [Andy, over 40 years old]

Those who viewed the medication as useful said that it had helped mainly in reducing anxiety, and that it had made thinking easier, especially in the acute crisis. One of them found medication important only at the early phase of the crisis, when it had made it possible to have contact with other people.

My level of fear was just so high! I mean, no normal person could ever sink so far down. I felt like I was at the bottom of a hundred canyons, and I was afraid of this real world as well as of my parents, and I’m quite certain that those therapists were not able to get anything out of me back then. Somehow those meds just eventually helped me to say something to them. I needed them only to get over the worst, and since then I have managed well without them. [Kate, 20-40 years old]

Out of 12 participants who received anxiolytics during the acute psychosis, two indicated that the anxiolytics had made them too tired.

## Discussion

This is first study exploring the retrospective experiences of first-episode psychosis treatment over the longer term, as undergone by persons who participated in the original Open Dialogue research projects in the Western Lapland catchment area. After a period of years from their initial crisis, most participants indicated that the important element in their mental healthcare for first-episode psychosis was the possibility to discuss difficult experiences openly, together with the fact that someone was showing an interest in an open-minded and non-judgemental manner. However, some participants were remembering that there had sometimes been too many people participating in the treatment meetings, and that the immediate home visits and inclusion of family members had not worked well in their situation. Regarding antipsychotics, even though many of those who had used them felt that the medication had reduced some of the distressing experiences during the acute crisis, many had also experienced disabling side-effects.

The main findings resemble earlier studies on first-person accounts of OD. In these studies most persons under OD-based treatment indicated that they were listened to and understood, although a minority had had mixed experiences regarding some aspects of the approach (e.g. Florence et al., 2021; Tribe et al., 2019; Wusinich et al., 2020). It nevertheless should be noted that, as already reported in earlier study (Bergström et al., 2019), in the majority of participants’ life stories treatment-related narratives were de-emphasized as compared to other themes; rather than focusing on mental health treatment, participants underlined the support from their close networks as well as their own actions in the gradual process of surviving from psychosis. This may be due to the long follow-up time, but also to the treatment approach itself, since the OD might shift the entire treatment process closer to “real-life”, by blurring the conventional roles of service users, family members, and service providers (Bergström, 2020). This, along with the fact that at the time OD was the standard approach to care in the region, might also explain why there were no comparisons between OD and other types of mental health treatment that have been expressed in some previous studies (e.g. Piippo, 2008; Tribe et al., 2019; Wusinich et al., 2020). The actual clinical significance of these findings merits further studies.

At a more general level, the overall results of this study align with earlier research on the common factor perspective, regarding the importance of therapeutic relationships and of collaborative care approaches (e.g. Byrne et al., 2010; Hansen et al., 2018). Moreover, participants’ criticisms of the mental health care treatment they received were relatively mild especially as compared to those expressed in some earlier studies. For example, a meta-synthesis (Griffiths et al., 2019) on first-person experiences of psychosis indicated ways in which participants described a range of unhelpful responses from health professionals. These increased their distress, making them feel dismissed and not treated as an individual. These elements did not appear in the interviews for the present study.

Nevertheless, it is important to be aware of the critical experiences related to some very basic elements of OD. For example, one participant reported challenges involving a family member who she identified as a signifiant source of her problems. Further research is needed into how family- and network-oriented approaches can be safely and effectively applied in these situations. Correspondingly, even if OD puts a clear emphasis on an immediate response, there is a risk of conducting it in a manner that does not sufficiently respect the unique situation of the person undergoing the crisis. The same would apply to the teamwork aspect, which is also one of the basic elements of OD. These make it clear how important it is to carefully consider each unique situation, including how the people in crisis wish to be met.

## Strength and Limitations

All cohort members living within a reasonable distance from research site were granted the opportunity to participate. Many had no recent contact with the mental health care system; there was a loss of potential participants, as expected. Moreover, as we were able to recruit directly only persons who were still receiving treatment, there was potential over-representation of participants with more severe symptomatology. Thus, the participants’ experiences do not necessarily constitute a valid representation of all the cohort members’ experiences.

Although the sample size was small and non-random, it can still be considered adequate for qualitative research, within which the interest lies in a phenomenological interpretation and in the subjectivity of experiences, rather than in reductive findings based on numerical analyses. In the future, the validity and generalizability of the findings could be further evaluated via case-to-case translation, which would involve applying findings from an inquiry encompassing a different group of people (Polit & Beck, 2010).

Beyond this, there were some other limitations that could reduce the validity of the main findings. First of all, the position held by the interviewer increased the risk of demand effect (Nichols & Maner, 2008). To minimize this, all the interviews were conducted by the first author, who had no role in the participants’ past or present treatment. To further minimize both subjectivity and demand bias the interview-protocol was minimally structured, with a main focus on open-ended questions regarding participants’ life stories.

Another limitation is that the participants were not systematically asked to review the transcriptions or analyses. Thus, some aspects might have been downplayed, or simply misunderstood, with the researchers’ own preconceptions affecting both the course of the interviews and the analysis of the data. In the future this kind of a bias could be compensated for by integrating member-checking procedures within the initial research protocol.

Even though the analyses were jointly discussed by all the authors, the initial analysis was done by the first author, and this could further increase the risk of subjectivity bias. Even though the first author had no role in implementing or developing OD and did not have any financial ties with past or present OD training programs, he had worked as a clinical psychologist in the Western Lapland region; this could have influenced the interpretation of the findings. Moreover, inclusion of members of the original OD team in the research group could further increase researcher allegiance bias (see e.g. Leykin & DeRubeis, 2009). These issues were recognized and openly discussed throughout the project.

## Conclusions

For the most part, participants from the Western Lapland catchment area had experienced their first-episode psychosis treatment under Open Dialogue-based services as helpful in managing a difficult life-situation. At the same time, a minority of those interviewed questioned the benefit of some characteristics of the approach. Overall, it appeared that dialogical responses to the crisis do have the potential to promote therapeutic relationships, and this may contribute to the improved mental health treatment outcomes reported in earlier studies (Bergström et al., 2018). Replications from other catchment areas are needed.

## Data Availability

Due to the confidential nature of this study, data of interviews is not available.
